# Melancholic-Like Behaviors and Circadian Neurobiological Abnormalities in Melatonin MT_1_ Receptor Knockout Mice

**DOI:** 10.1093/ijnp/pyu075

**Published:** 2015-01-28

**Authors:** Stefano Comai, Rafael Ochoa-Sanchez, Sergio Dominguez-Lopez, Francis Rodriguez Bambico, Gabriella Gobbi

**Affiliations:** Neurobiological Psychiatry Unit, Department of Psychiatry, McGill University and McGill University Health Center, Montréal, QC, Canada (Drs Comai, Ochoa-Sanchez, Dominguez-Lopez, Bambico, and Gobbi).

**Keywords:** corticosterone, daily mood variations, melancholic depression, monoamines, MT1 receptors, norepinephrine, serotonin, circadian rhythm

## Abstract

**Background:**

Melancholic depression, described also as endogenous depression, is a mood disorder with distinctive specific psychopathological features and biological homogeneity, including anhedonia, circadian variation of mood, psychomotor activation, weight loss, diurnal cortisol changes, and sleep disturbances. Although several hypotheses have been proposed, the etiology of this disorder is still unknown.

**Methods:**

Behavioral, electrophysiological and biochemical approaches were used to characterize the emotional phenotype, serotonergic and noradrenergic electrical activity, and corticosterone in melatonin MT1 receptor knockout mice and their wild type counterparts, during both light and dark phases.

**Results:**

Melatonin MT_1_ receptor knockout mice have decreased mobility in the forced swim and tail suspension tests as well as decreased sucrose consumption, mostly during the dark/inactive phase. These mood variations are reversed by chronic treatment with the tricyclic antidepressant desipramine. In addition, MT_1_ receptor knockout mice exhibit psychomotor disturbances, higher serum levels of corticosterone the dark phase, and a blunted circadian variation of corticosterone levels. *In vivo* electrophysiological recordings show a decreased burst-firing activity of locus coeruleus norepinephrine neurons during the dark phase. The circadian physiological variation in the spontaneous firing activity of high-firing neuronal subpopulations of both norepinephrine neurons and dorsal raphe serotonin neurons are abolished in MT_1_ knockout mice.

**Conclusions:**

These data demonstrate that melatonin MT_1_ receptor knockout mice recapitulate several behavioral and neurobiological circadian changes of human melancholic depression and, for the first time, suggest that the MT_1_ receptor may be implicated in the pathogenesis of melancholic depression and is a potential pharmacological target for this mental condition.

## Introduction

Melancholia, also described as endogenous, endogenomorphic, autonomous, type A, psychotic, and typical depression (Parker et al., 2010), is a mood disorder with specific psychopathological symptoms, including disturbances in affect, diurnal variation with mood generally worse in the morning, anhedonia, psychomotor retardation or agitation, cognitive impairment, and neurobiological/somatic impairments manifested as loss of weight, hypercortisolemia, and sleep disturbances, mainly at the level of rapid eye movement sleep (REMS; Rush and Weissenburger, 1994; Armitage, 2007). Melancholic patients respond better to broad-action tricyclic antidepressants than to narrow-action antidepressants (Perry, 1996), and their response to placebo, psychotherapy, or even social intervention is very poor (Brown, 2007).

Few animal models of melancholic depression have been proposed (Hill and Gorzalka, 2005; Kato et al., 2008; Shen et al., 2010), but their attempts to reproduce the whole complex symptomatology of the disease, in particular the diurnal variations of mood, failed.

Diurnal mood variations are indeed the hallmark of melancholic depression (Parker et al., 2010), and differentiate melancholic versus non-melancholic depression (Duncan, 1996). They have been linked to a dysfunction in circadian rhythms that are driven by the suprachiasmatic nucleus (SCN). The activity of the SCN is regulated by the neurohormone melatonin (MLT), which acts through its two G protein–coupled receptors, MT_1_ and MT_2_ (Liu et al., 1997; Jin et al., 2003; Dubocovich, 2007). Previous literature has suggested a link between MLT and melancholic depression. Brown et al. (1985) reported lower nocturnal MLT levels in melancholic patients than in controls, and Fountoulakis et al. (2001) found that melancholic patients had lower 23.00h MLT blood levels in comparison to atypical, somatic syndrome, or “undifferentiated” depressed patients. Melatonin MT_1_ receptor knockout (MT_1_^-/-^) mice showed altered REMS and sleep architecture (Comai et al., 2013), increased immobility in the forced swim test, and deficits in sensorimotor gating (Weil et al., 2006). Altogether, these findings led us to extensively investigate the psychobiological phenotype of MT_1_^-/-^ mice as a possible model of melancholic depression using multiple behavioral and biological tests and *in vivo* electrophysiology recording of serotonin (5-HT) and norepinephrine (NE) neurons, whose neurotransmission was impaired in melancholic depression (Pier et al., 2004; Malhi et al., 2005). Nonetheless, we tried to reverse their depressive-like phenotype using the tricyclic antidepressant desipramine. To detect diurnal changes observed in melancholic patients, all experiments were performed during both the light and dark phases.

## Materials and Methods

### Animals

Adult (2–4 month-old) male C3H/HeN mice were purchased from Charles River. C3H/HeN MT_1_^-/-^ mice (Liu et al., 1997) were purchased from David Weaver (University of Massachusetts Medical School), and age-matched mice born in our facility were also used. Mice were kept under standard laboratory conditions (12h light/dark cycle, lights on at 07:30h; temperature at 20±2°C) with food and water *ad libitum*, and were used and handled in accordance with the guidelines of McGill University and the Canadian Institutes of Health Research for animal care and scientific use. Light phase experiments were conducted between 14:00 and 17:00h, whereas dark phase experiments between 2:00 and 5:00h.

### Treatment

Desipramine (10mg/kg, Sigma-Aldrich) was dissolved in 0.9% NaCl and injected intraperitoneally (0.1ml) once daily for 20 days.

### Behavior

We used 7–11 mice per genotype and phase of the day. Given the high number of behavioral tests to be performed, we employed three different groups of animals per genotype and phase of the day: one group underwent the open field, forced swim, and tail suspension tests; a second group was used in the elevated plus maze and novelty suppressed feeding tests; and the third group was evaluated in the sucrose preference test only. For animals of groups 1 and 2, a 7-day interim period was interlaced between tests to minimize the stress and mood effects of one experiment carrying over to the next. For habituation, mice were placed in the behavioral room 1h prior to the experimental session. The apparatus was cleaned before each session using a 70% alcohol solution and paper towel. Behaviors were recorded, stored, and analyzed using an automated behavioral tracking system (Videotrack, View Point Life Science) equipped with infrared light-sensitive CCD cameras. Light phase experiments were conducted using standard room lighting (350lx) and a white lamp (100W), and dark phase experiments using infrared light-emitting diodes and a lamp with a red light bulb (8 lux).

### Open Field Test

Mice were singly placed at the corner of a white-painted open field arena (40×40×30cm) and their behavior was recorded for 20min. Frequency and total duration of central zone (20×20cm) visits and total locomotor activity were analyzed (Bambico et al., 2010).

### Novelty-Suppressed Feeding Test

Mice were food-deprived for 48h and then individually placed in the corner of an open arena (40×40×30cm) containing 3 pellets of lab chow in the middle. The latency to initiate feeding was measured. Animals were then returned to the home cage, in which 3 pellets of food where placed in the center. The home cage feeding latency was noted (Bambico et al., 2010).

### Elevated Plus Maze Test

The plus maze (50cm off the floor) was made of white Plexiglass with two open arms (30×5cm) and two arms of the same size enclosed by walls (15cm) which converge perpendicularly into a central platform (5×5cm). Mice were singly placed in the central platform facing the open arm, and their behavior was recorded for 5min. The following measures were collected: time spent in the open and closed arms, frequency of open and closed arm entries, and frequency and total duration of head dips beyond the borders of the open arms (Bambico et al., 2010).

### Forced Swim Test

The test was conducted according to Porsolt et al. (1977). Mice were individually placed in a Plexiglas cylinder (20cm diameter, 50cm high) containing 20cm water (25°C), from which they could not escape. The experiment lasted 6min and the duration and frequency of immobility during the last 4min were analyzed.

### Tail Suspension Test

Mice were individually suspended from a lever by adhesive tape placed approximately 1cm from the tip of the tail. Duration and frequency of immobility were determined for 6min (Bambico et al., 2010).

### Sucrose Preference Test

Mice were individually housed 3 days before the beginning of the test. They were then trained for 3 days to consume water from two bottles. During these 3 days, the two bottles containing water were replaced for 1h a day with two bottles filled with a 2% (w/v) sucrose solution. Next, mice were subjected to a 48h procedure during which they were allowed to discriminate and select between 2 drinking bottles, one containing water and the other the sucrose solution. To avoid conditioned place preference learning, the position of the bottles was switched at the middle of the light and dark phases. Bottles were weighed at the onset of the light and dark phases in order to measure separately the sucrose preference for each phase of the day. The sucrose preference (%) was determined as follows: sucrose solution intake (g)/total fluid intake (g) × 100.

### Electrophysiology

*In vivo* single-unit extracellular recordings of dorsal raphe nucleus (DRN) 5-HT and locus coeruleus (LC) NE neurons were performed following well-validated procedures (Gobbi et al., 2005; Bambico et al., 2010; Bambico, 2010) in our lab and are detailed in Supplementary Figure 1. Briefly, mice were anesthetized and placed in a stereotaxic frame. The stereotaxic brain coordinates of the DRN and LC were in agreement with the Paxinos and Franklin (2001) atlas. Spontaneous electrical activity of single cells was recorded using single-barreled glass micropipettes. The analog signal was converted into a digital signal via a 1401 Plus interface (CED) and analyzed off-line using Spike2 version 5.20 (CED). The recording site was marked for later histological verification.

### Determination of Corticosterone Serum Levels

All animals were sacrificed by decapitation at the same time behavioral and electrophysiological experiments were conducted (light phase, 14:00h; dark phase, 02:00h). Trunk blood was collected within 30 s after the animal’s removal from the cage. Corticosterone serum levels were analyzed using the DetectX Corticosterone Enzyme Immunoassay kit (K-014-H1, Arbor Assays).

### Statistical Analysis

Data are reported as mean ± standard error of the mean. Data analysis was performed using SigmaPlot 11 and SPSS 17. When normality and variance homogeneity were satisfied, two-way analyses of variance (ANOVA) or two-way ANOVA for repeated measures followed by Student-Newman-Keuls post hoc comparisons were used, employing the factors genotype and phase of the day. Three-way ANOVA was used to assess the effects of desipramine. Student’s *t*-test was used to compare weight differences between genotypes. Differences between subgroups of 5-HT and NE firing neurons were measured using the Kruskal–Wallis ANOVA on ranks followed by Dunn’s method. Genotype differences in the open field test (OFT) parameters were analyzed using a regression analysis controlling for the factor time. Fisher’s exact test was employed to determine genotype differences between bursting and non-bursting LC-NE neurons. A *p-*value *<* 0.05 was considered significant.

## Results

### MT_1_^-/-^ Mice Display a Depressive–Like Phenotype and Anhedonia

**Figure 1.**
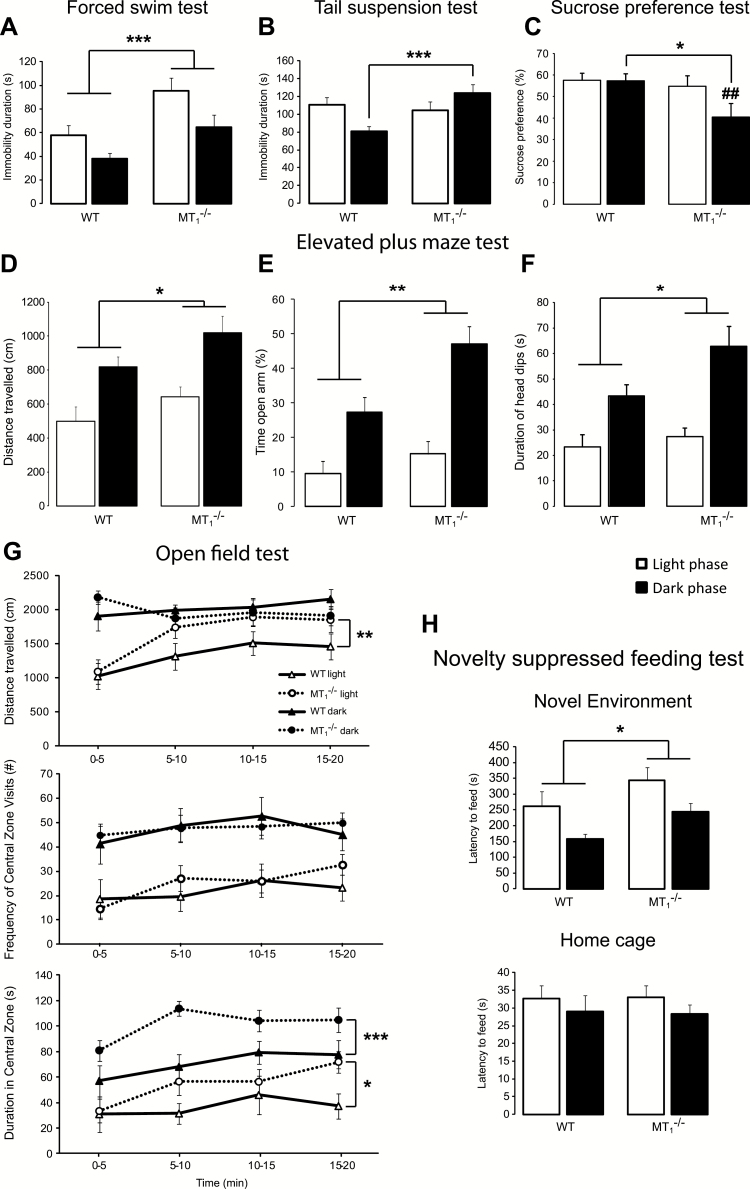
MT_1_^-/-^ mice displayed depressive-like behavior and psychomotor disturbances. MT_1_^-/-^ mice showed increased immobility time in the forced swim test (A) and in the tail suspension test (B). (C) The sucrose preference was decreased during the dark phase and was also affected by the phase of the day in MT_1_^-/-^ mice. MT_1_^-/-^ mice exhibited increased locomotion (D), greater % of time spent in the open arm (E), and longer time spent head dipping (F) in the elevated plus maze test. (G) Locomotor activity in the open field test is increased in MT_1_^-/-^ mice during the light phase. The time spent in the center of the open field is higher in MT_1_^-/-^ mice during both the light and the dark phases. (H) MT_1_^-/-^ mice showed increased latency to eat in a new environment but not in the home cage in the novelty suppressed feeding test. Results are given as mean ± standard error of the mean. n = 10 per genotype in the light phase and n = 11 per genotype in the dark phase. *p < 0.05 and ***p < 0.001 MT_1_^-/-^ vs. WT mice; ##*p* < 0.01 light vs. dark phase, two-way ANOVA, followed by Student-Newman-Keuls post hoc test. In the open field test (G), *p < 0.05, **p < 0.01, ***p < 0.001 MT_1_^-/-^ vs. WT mice, with regression analysis controlling for the factor time.

In the forced swim test (FST) and tail suspension test (TST; Figure 1), MT_1_^-/-^ mice showed a depressive-like phenotype when compared to wild-type controls (WT). In the FST (Figure 1A), MT_1_^-/-^ mice spent more time immobile than WT (effect of genotype: F_1,38_ = 12.46, *p* = 0.001; phase of the day: F_1,38_ = 7.74, *p* = 0.008; no interaction genotype x phase of the day). In the TST (Figure 1B), the duration of immobility was longer in MT_1_^-/-^ than in WT mice during the dark phase only (*p* = 0.006; interaction: F_1,38_ = 5.36, *p* = 0.026). The sucrose preference (Figure 1C), a measure of anhedonia, was reduced in MT_1_^-/-^ compared to WT mice during the dark phase only (*p* = 0.017, interaction: F_1,38_ = 6.37, *p* = 0.021). Interestingly, while no effect due to the phase of the day was observed in WT, in MT_1_^-/-^ mice the sucrose preference was lower during the dark than during the light phase (*p* < 0.002). In the novelty-suppressed feeding test (NSFT; Figure 1H), the latency to eat in a new environment was longer in MT_1_^-/-^ than in WT mice (genotype: F_1,38_ = 6.07, *p* = 0.018; phase of the day: F_1,38_ = 8.95, *p* = 0.005; no interaction). No differences were observed for the latency to eat in the home cage.

### MT_1_^-/-^ Mice Exhibit Hyperactivity and Increased Time in the Center of OFT and in the Open Arm of EPMT

Regression analysis, testing for genotype and controlling for the factor time, showed that in the OFT (Figure 1G) MT_1_^-/-^ mice covered a longer distance than WT during the light phase (*p* = 0.005). While the number of entries in the center of the OF was not affected by genotype, the time spent in the center of the open field was higher in MT_1_^-/-^ than in WT mice during both light (*p* = 0.015) and dark (*p* < 0.001) phases. In the elevated plus maze test (EPMT; Figure 1D–F), the distance traveled during the 5min session by MT_1_^-/-^ mice was longer than that covered by WT (genotype: F_1,38_ = 419, *p* = 0.047; Figure 1D). The distance traveled was longer during the dark phase compared to the light phase (phase of the day: F_1,38_ = 23.50, *p <* 0.001). The percentage of time spent in the open arm was greater in MT_1_^-/-^ compared to WT mice (*p =* 0.006) and longer during the dark phase (genotype: F_1,38_ = 8.64, *p* = 0.006; phase of the day: F_1,38_ = 32.21, *p <* 0.001; no interaction). The total duration of head dips was increased in MT_1_^-/-^ compared to WT mice (genotype: F_1,38_ = 4.19, *p* = 0.047) and longer during the light than during the dark phase (phase of the day: F_1,38_ = 20.78, *p <* 0.001).

### Chronic Treatment with Desipramine Reverses the Depressive-Like Phenotype of MT_1_^-/-^ Mice

**Figure 2.**
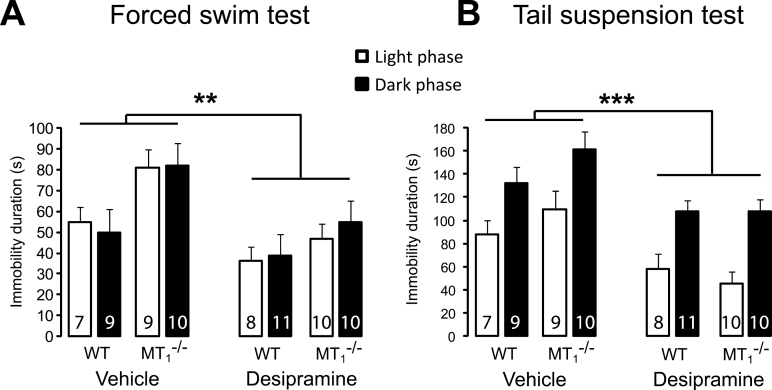
Chronic antidepressant treatment with desipramine reverses depressive-like behavior of MT_1_^-/-^ mice. (A and B) Desipramine reduced the immobility time in the forced swim test (A) and in the tail suspension test (B) in WT and MT_1_^-/-^ mice. Results are given as mean ± standard error of the mean. The number of animals per genotype and phase of the day is reported within the bar. *p < 0.01 and **p < 0.001 desipramine vs. vehicle, three-way ANOVA followed by Student-Newman-Keuls post hoc test.

After 20 days of treatment with desipramine, WT and MT_1_^-/-^ mice underwent the FST and the TST (Figure 2). In the FST (Figure 2A), desipramine reversed the differences of the duration of immobility between WT and MT_1_^-/-^ that were observed in animals receiving vehicle. Three-way ANOVA (genotype x treatment x phase of the day) indicated an effect of treatment (F_1,66_ = 10.36, *p =* 0.002) and an effect of genotype (F_1,66_ = 8.93, *p =* 0.004), with no interaction. Similarly, in the TST (Figure 2B) desipramine produced a significant decrease of immobility in both WT and MT_1_^-/-^ mice (treatment: F_1,66_ = 18.74, *p <* 0.001; phase of the day: F_1,66_ = 33.28, *p <* 0.001; no interaction), reinstating a normal behavioral phenotype in MT_1_^-/-^ mice. In order to rule out possible false-negative or false-positive results in the FST and TST due to the effect of desipramine on motor activity, mice were tested for 10min in the OFT prior to the FST (Figure S1 in Supplementary Material). Desipramine significantly reduced the locomotor activity of both WT and MT_1_^-/-^ mice, thus confirming its antidepressant-like properties (increased swimming, decreased immobility) in both behavioral paradigms of depression (treatment: F_1,66_ = 11.27, *p <* 0.001; phase of the day: F_1,66_ = 80.98, *p <* 0.001; no interactions).

### LC NE Neural Activity is Reduced in MT_1_^-/-^ Mice

**Figure 3.**
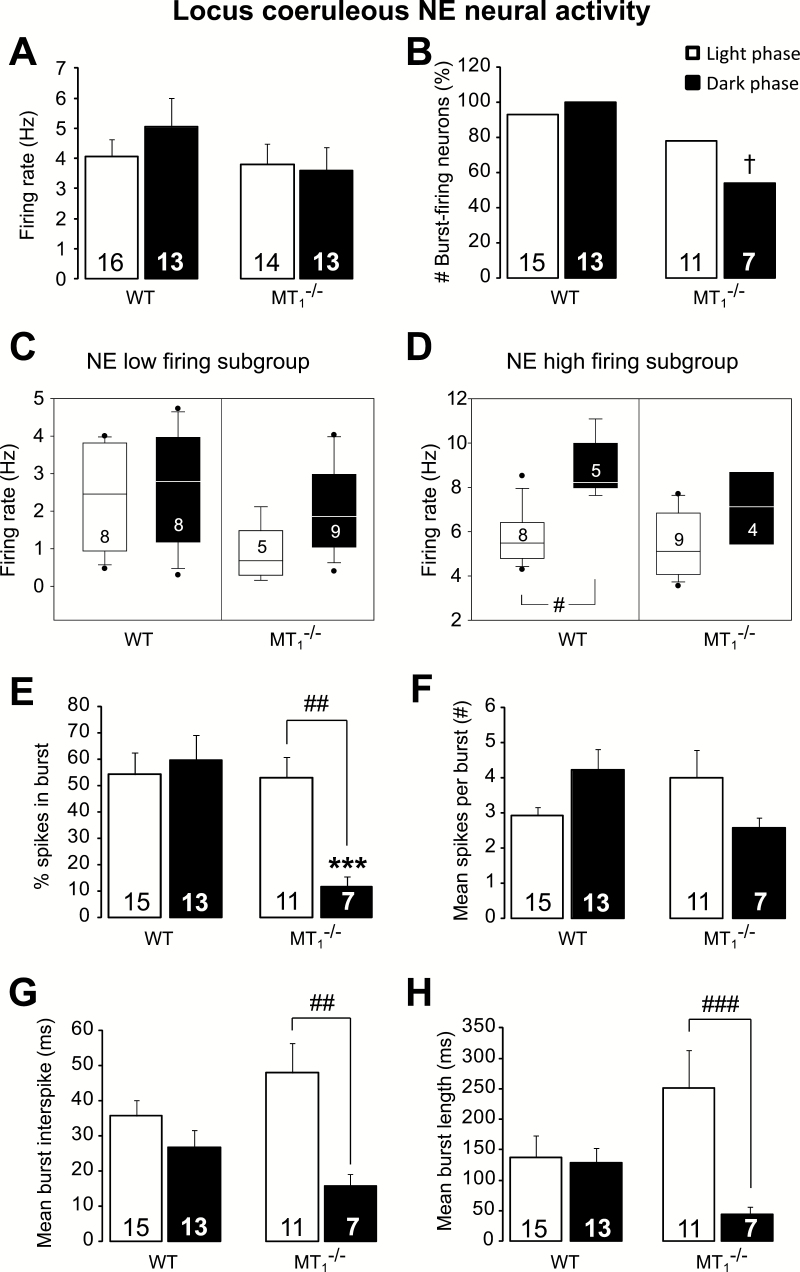
Locus coeruleus norepinephrine (NE) burst activity is reduced in MT_1_^-/-^ mice. (A) Mean NE firing activity in WT and MT_1_^-/-^ mice during the light and the dark phases. (B) The percentage of locus coeruleus NE neurons discharging in bursts is lower in MT_1_^-/-^ mice during the dark phase. Boxplot representation of the low (C) and high (D) NE single-spike firing subgroups. Neural activity of the NE high-firing subgroup does not increase during the dark/active phase in MT_1_^-/-^ mice (D). Horizontal lines within boxes, boxes, and error bars respectively represent the median, the 25^th^, and 75^th^ percentiles and the 10^th^ and 90^th^ percentiles. The outliers are displayed as individual points. #*p* < 0.05, Kruskal–Wallis analyses of variance on ranks followed by Dunn’s method. (E–H) NE neuronal firing pattern in WT and MT_1_^-/-^ mice during the light and dark phases. The percentage of spikes in burst (E), the mean burst interspike (G), and the mean burst length (H) are lower during the dark compared to the light phase in MT_1_^-/-^ mice only. Results are given as mean ± standard error of the mean and the number of recorded neurons is indicated at the bottom of each column. †*p* < 0.05 MT_1_^-/-^ vs. WT mice, Fisher’s exact test. *p < 0.001 MT_1_^-/-^ vs. WT mice; ##*p* < 0.01 light vs. dark phase, two-way ANOVA, followed by Student-Newman-Keuls post hoc test.

No difference between genotypes was found in the spontaneous firing rate of LC NE neurons (Figure 3A). Conversely, important changes were detected regarding the neural burst activity. Fisher’s exact test showed that during the dark phase, the percentage of bursting-firing neurons is lower in MT_1_^-/-^ than in WT mice (*p* = 0.015, Figure 3B). An example of a LC NE neural recording from WT and MT_1_^-/-^ mice is reported in Figure S2 in Supplementary Material.

According to our previous study (Bambico et al., 2010), a *K*-means cluster analysis allowed us to identify the clusters of NE neurons with low and high firing activities for each genotype and phase of the day (Figure 3C and D). No differences were observed regarding the subgroup of LC neurons with low firing activity (Figure 3C) but, interestingly, Kruskal-Wallis one-way ANOVA on ranks [H(3) = 10.33; *p* = 0.016] showed that the neural activity of LC NE neurons belonging to the high firing subgroup was higher during the dark than during the light phase in WT mice (8.22 Hz [25th/75th percentile, 4.76/6.51 Hz] vs. 5.48 Hz [7.86/10.35]; *p* < 0.05, q = 3.00; Figure 3D). Importantly, the physiological light/dark difference was abolished in MT_1_^-/-^ mice.

Figure 3E–H reports the analysis of the LC NE neuronal burst activity. The percentage of spikes in burst (Figure 3E) was lower in MT_1_^-/-^ than in WT mice (*p =* 0.001) and changed according to the phase of the day in MT_1_^-/-^ mice only (interaction: F_1,42_ = 6.78, *p =* 0.013; genotype: F_1,42_ = 7.45, *p =* 0.009; phase of the day: F_1,42_ = 3.94, *p =* 0.05).

A tendency to fewer spikes per burst during the dark phase was found in MT_1_^-/-^ compared to WT mice (*p =* 0.067; interaction: F_1,42_ = 5.55, *p =* 0.023; Figure 3F). No genotype differences and an interaction genotype per phase of the day were found for the mean burst interspike (interaction: F_1,42_ = 3.71, *p =* 0.060) and the mean burst length (interaction: F_1,42_ = 4.64, *p =* 0.037). Very importantly, while the two parameters did not vary according to the phase of the day in WT animals, in MT_1_^-/-^ mice they were both higher during the light than during the dark phase (*p =* 0.002 and *p =* 0.007, respectively; Figure 3G and H).

### DRN 5-HT Neural Activity is Altered in MT_1_^-/-^ Mice

**Figure 4.**
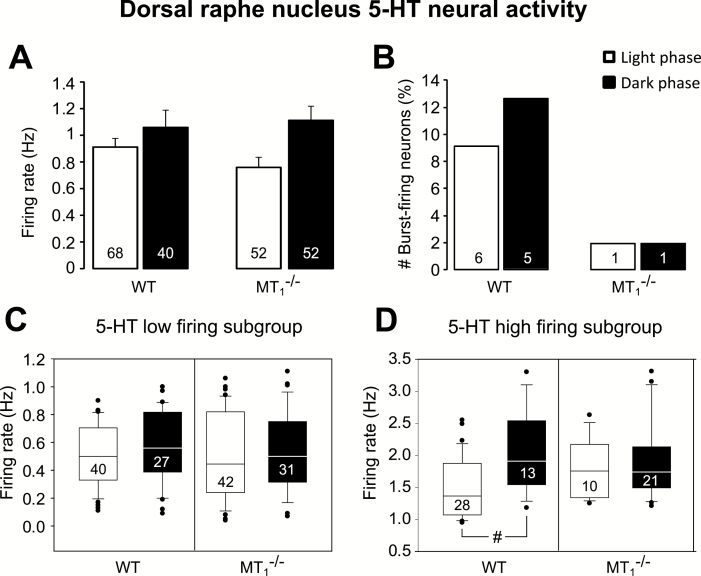
Dorsal raphe serotonergic high-firing activity in MT_1_^-/-^ mice does not change according to the phase of the day. (A) Mean 5-HT firing activity in WT and MT_1_^-/-^ mice during the light and the dark phases. (B) Percentage of dorsal raphe nucleus 5-HT neurons discharging in burst. Results are given as mean ± standard error of the mean. The total number of neurons per genotype and phase of the day is reported within the bar. Boxplot representation of the low (C) and high (D) 5-HT single-spike firing subgroups. Neural activity of the 5-HT high firing subgroup does not increase during the dark/active phase in MT_1_^-/-^ mice (D). Horizontal lines within boxes, boxes, and error bars respectively represent the median, the 25^th^, and 75^th^ percentiles and the 10^th^ and 90^th^ percentiles. The outliers are displayed as individual points. #*p* < 0.05, Kruskal–Wallis analysis of variance on ranks followed by Dunn’s method.

No differences in the DRN 5-HT firing rate between WT and MT_1_^-/-^ mice were observed (Figure 4A). Importantly, DRN 5-HT firing activity was higher during the dark than during the light phase (phase of the day: F_1,208_ = 6.18, *p =* 0.014). Among all 5-HT neurons recorded in MT_1_^-/-^ mice, only one per phase of the day was found to discharge in bursts. On the contrary, in WT mice we found that 8.8% and 12.5% of the neurons were discharging in bursts during the light and the dark phase, respectively (Figure 4B). Due to the limited number of 5-HT bursting neurons in MT_1_^-/-^ mice, statistical comparisons versus WT could not be performed. However, a clear reduction in 5-HT neurons discharging in bursts was observed in MT_1_^-/-^ mice. An example of a DRN 5-HT neural recording from WT and MT_1_^-/-^ mice is reported in Figure S3 in Supplementary Material.

Two subpopulations of 5-HT neurons, one with low and another with high firing activity, could be identified using *K*-means cluster analysis (Bambico, Cassano, et al., 2010; Figure 4C and D). No difference in the firing rate between the subgroups with low 5-HT firing neurons was found (Figure 4C). Conversely, in analyzing the subgroups with high firing 5-HT neurons (Figure 4D), the median value during the dark phase was higher than during the light phase in WT mice (Kruskal-Wallis one-way ANOVA on ranks: H(3) = 11.03, *p* = 0.012; 1.91 Hz [25th/75th percentile, 1.51/2.62 Hz] vs. 1.36 Hz [1.04/1.87]; *p* < 0.05, q = 4.06). Remarkably, such diurnal difference was abolished in MT_1_^-/-^ mice (Figure 4D).

### Corticosterone Serum Levels are Higher in MT_1_^-/-^ Mice During the Dark Phase

**Figure 5.**
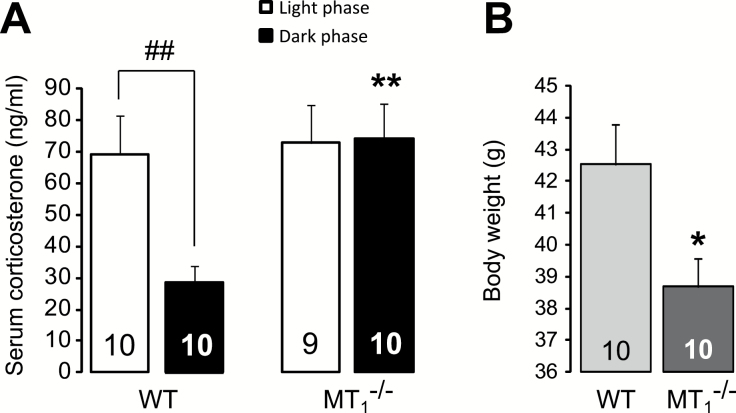
MT_1_^-/-^ mice displayed altered serum corticosterone levels and reduced body weight. (A) Serum corticosterone levels are higher during the dark phase and do not change according to the phase of the day in MT_1_^-/-^ mice. (B) 5 month-old MT_1_^-/-^ mice showed decreased body weight. **p < 0.01 MT_1_^-/-^ vs. WT mice; ##*p* < 0.01 light vs. dark phase, two-way ANOVA, followed by Student-Newman-Keuls post hoc test. *p < 0.05 MT_1_^-/-^ vs. WT mice, student t-test.

The serum levels of corticosterone (Figure 5A) were found to be higher in MT_1_^-/-^ than in WT mice during the dark phase (*p =* 0.003; interaction: F_1,35_ = 4.14, *p =* 0.049; genotype: F_1,35_ = 5.68, *p =* 0.023; phase of the day: F_1,35_ = 3.57, *p =* 0.067), and more importantly, the physiological diurnal variation of cortisol was disrupted in MT_1_^-/-^ mice.

### MT_1_^-/-^ Mice Weigh Less than WT

5 month-old MT_1_^-/-^ mice displayed a reduced body weight when compared to similar age WT (-9.07%; student’s *t*-test, *t* = 2.50, 18 df, *p* = 0.022; Figure 5B).

## Discussion

We have examined the behavioral characteristics, the serotonergic and norepinephrine neural activities, and the corticosterone levels of MT_1_^-/-^ mice. Our results suggest that MT_1_^-/-^ mice may be considered an animal model of human melancholic depression. Indeed, they display (1) anhedonia, (2) depressive-like phenotype with diurnal variations, (3) reduced body weight, (4) hyperlocomotion, (5) changes in the monoaminergic neural activity, and (6) altered serum levels of corticosterone with disrupted circadian variation, all features that are core symptoms of melancholia (Table 1). Remarkably, the depressive-like symptoms manifested by MT_1_^-/-^ mice were reversed by chronic treatment with desipramine. Similar to melancholic patients (Parker et al., 2010), MT_1_^-/-^ mice also exhibited REMS disturbances and reduced power of the delta band of non-REMS, a measure linked to the depth of sleep (Comai et al., 2013).

**Table 1.** A Comparison Between the Psychological and Neurobiological Symptoms of Melancholic Depression and the Phenotype of MT1 Receptors Knockout Mice.

Symptoms of melancholic depression (Parker et al., 2010)	MT_1_ receptors knockout mice
Anhedonia	Anhedonia (↓ sucrose preference)
Depression	Depression-like behavior (↑ immobility in FST and TST; ↑ latency to eat in NSFT)
Weight loss	↓ Weight
Psychomotor disturbances (agitation or retardation)	Hyperlocomotion (↑ locomotion in OFT), disinhibition (↑ open arm time and entries in EPMT, ↑ head dips in EPMT)
Circadian variation of mood	behavioral light/dark differences
Hypercortisolemia	↑ corticosterone serum levels during the dark phase, no light/dark differences in corticosterone levels
Disturbance in sleep architecture especially at the level of REMS	↓ REMS duration, ↓ NREMS EEG delta power and REMS EEG theta power (36)
Monoamine activity alterations	↓ DRN 5-HT and LC NE neuronal bursts activity; altered light/dark firing pattern of DRN 5-HT and LC NE neurons
Genetic causes	Genetic inactivation of MT_1_ receptors

↑ = increase; ↓ = decrease. DRN, dorsal raphe nucleus; EEG, electroencephalographic; EPMT, elevated plus maze test; FST, forced swim test; LC, locus coeruleus; NE, norepinephrine; NREMS, non-REMS; NSFT, novelty-suppressed feeding test; OFT, open field test; REMS, rapid eye movement sleep; TST, tail suspension test.

Weil et al. (2006) confirmed that MT_1_^-/-^ mice had increased immobility in the FST and an impaired prepulse inhibition response, a neurobiological sign often correlated to psychotic-like symptoms. Notably, melancholic depression can also include psychotic symptoms (Caldieraro et al., 2013).

The results obtained in the OFT (decreased thigmotaxis) and in the EPMT (increased % of time spent in the open arm) suggest an “anxiety-resistant” phenotype of MT_1_^-/-^ mice, while the increased latency to feed in the NSFT may suggest an anxiogenic-like phenotype. Nevertheless, in the OFT and EPMT, MT_1_^-/-^ mice showed increased locomotor activity, which may be a bias when evaluating anxiety-related parameters such as tigmotaxis and percentage of entries into the open arms, respectively (Dawson and Tricklebank, 1995). Therefore, the apparent “anxiety-resistant” phenotype of MT_1_^-/-^ mice could instead be the result of a disinhibitory/hyperactive behavior rather than a state of hypo-anxiety. For this reason, the NSFT represents, in this case, the most reliable test for anxiety in MT_1_^-/-^ mice. Indeed, even though in the novel arena knockout mice showed increased locomotor activity and decreased thigmotaxis, the latency to feed was significantly longer, further validating their anxiogenic-like phenotype. In keeping with the anxiogenic behavior in MT_1_^-/-^ mice, melancholic patients usually display increased levels of anxiety (Day and Williams, 2012). However, Parker et al. (2013) found a lower prevalence of anxiety disorders in melancholic than in non-melancholic patients, and therefore, the issue of anxiety in melancholic depression is complex.

Finally, even if the interpretation of the results obtained in the tests of anxiety may be arguable, the complex phenotype emerging from the EPMT, OFT, and NSFT perfectly reflect the multifaceted manifestations of anxiety observed in melancholic patients (Day and Williams, 2012; Parker et al., 2013). MLT, through MT_1_ and MT_2_ receptors, controls the activity of the SCN; in particular, MT_1_ receptors are involved in the acute inhibitory effect of MLT on the SCN firing rate (Liu et al., 1997), contributing to the control of circadian rhythms. Importantly, SCN projects to the locus coeruleus (Aston-Jones et al., 2001) and dorsal raphe (Deurveilher and Semba, 2005), influencing the rhythms of noradrenaline and serotonin, respectively.

NE and 5-HT are involved in the pathophysiology of depression (Gobbi and Blier, 2005), and our results show that the two monoaminergic neurotransmissions are significantly altered after genetic inactivation of MT_1_ receptors. In keeping with increased LC NE and DRN 5-HT firing activity during the dark/active phase (Aston-Jones et al., 2001; Ursin, 2002), the spontaneous firing rate of the high-firing subgroup of both monoamines was higher during the dark than during the light phase in control animals. Remarkably, this light/dark difference was abolished in MT_1_^-/-^ mice, suggesting a disruption of the diurnal pattern of monoaminergic electrical activity in these animals.

Moreover, MT_1_^-/-^ mice showed LC NE decreases in bursts-firing parameters during the dark/active phase and, notably, the increased LC NE burst firing activity is related to the release of the neurotransmitter in the terminal area (Florin-Lechner et al., 1996) and to antidepressant-like activity (Gobbi et al., 2007). One may hypothesize that the decrease in NE burst activity can be related to a depressive-like behavior.

Nonetheless, DRN 5-HT burst activity was also presumably reduced, even if the low number of bursting neurons found in this sample did not allow us to reach a significant statistical difference; a correlation has been well established between 5-HT burst activity, 5-HT release, and antidepressant-like activity (Gartside et al., 2000; Gobbi et al., 2005). NE and 5-HT neurotransmissions are also important regulators of the sleep/wake cycle and behavior (Aston-Jones et al., 2001; Ursin, 2002), and the NE and 5-HT neural impairments observed in knockout mice were paralleled by several behavioral and circadian impairments. Remarkably, the greater behavioral differences between WT and MT_1_^-/-^ mice were found during the active/dark phase, when the burst-firing activity of both monoamines was also reduced.

Most of the available treatments for depression target the monoaminergic systems, and melancholic patients have a greater response rate to old tricyclic antidepressants or monoamine oxidase inhibitors than to selective serotonin reuptake inhibitors (Perry, 1996). In agreement, a chronic treatment with desipramine was able to reverse the depressive-like phenotype of MT_1_^-/-^ mice, most likely (1) decreasing beta adrenergic neurotransmission in the hippocampus and affecting differentially the sensitivity and the response of somatodendritic and terminal α_2_-autoreceptors (Lacroix et al., 1991); and (2) increasing the responsiveness of postsynaptic hippocampus and ventral lateral geniculate neurons to 5-HT (Blier and de Montigny, 1994).

Even though desipramine is more selective for NE than for 5-HT (Frazer, 2001), it is not possible to disentangle whether the behavioral recovery observed in these knockout mice was due to NE, 5-HT, or both of them. Further experiments employing pharmacological agents selective for each monoamine (e.g., one selective serotonin reuptake inhibitor and one norepinephrine reuptake inhibitor) may help to address this question.

The other important biological feature that distinguishes melancholic versus non-melancholic depression is the sustained activation of the hypothalamic–pituitary–adrenal axis, which results in hypercortisolism and altered response to the dexamethasone suppression test (Roy et al., 1985; Parker et al., 2010). In keeping with this clinical evidence, MT_1_^-/-^ mice showed both increased serum corticosterone levels during the dark/active phase and blunted physiological circadian diurnal fluctuation, confirming the disturbances of the hypothalamic–pituitary–adrenal activity.

The role of MLT and its receptors in mood is not yet elucidated, and treatment with MLT alone does not seem to be an effective antidepressant strategy in humans (Carman et al., 1976; Quera Salva et al., 2011). Although few studies have associated MT_2_ receptors to depression (for review see Comai and Gobbi, 2014), the present data strongly underlines a role for MT_1_ receptors in the etiology of depression, in particular of melancholic depression. In agreement with this hypothesis, a specific increase of MT_1_ receptors in the SCN of depressive patients was recently found in a post-mortem study (Wu et al., 2013). Remarkably, the antidepressant agomelatine has a higher affinity for MT_1_ (*K*_*i*_ = 6.15x10^-11^ M) than for MT_2_ (2.68x10^-10^ M) or 5-HT_2c_ (IC_50_ = 2.7x10^-7^ M) receptors (de Bodinat et al., 2010), even though its antidepressant activity seems related to its multi-activity on MT_1_, MT_2_, 5-HT_2c_, and 5-HT_2b_ receptors (Chenu et al., 2014).

Here, we have reported for the first time that genetic inactivation of MT_1_ receptors in mice yield a phenotype that mimics several of the features of melancholic depression. Animal models of depression have proven to be of considerable value in elucidating pathophysiological mechanisms of disease and for developing novel treatments, but very few were able to reproduce the whole human symptomatology, especially regarding melancholic depression and its diurnal variations. Similarities between the behavioral responses of gamma-aminobutyric acid-type A receptor (Shen et al., 2010), CB_1_ receptor (Hill and Gorzalka, 2005), or Wfs1 (Kato et al., 2008) knockout mice and symptoms of melancholic depression have been reported. Unfortunately, none of these animal models were able to mimic the light/dark variation of symptoms observed in melancholic patients, and furthermore, they only displayed a few of the core symptoms of the disease. Several studies suggested that an impaired circadian system, regulated by a network of “clock genes,” contributes to the etiology and symptomatology of mood disorders, highlighting an association between mutations at the level of clock genes such us *Clock* or *Per1-3* and variations in mood (McCarthy and Welsh, 2012; Bunney and Bunney, 2013). But clock gene mutant animals also failed in capturing the full spectrum of symptoms shown by patients with mood disorders.

Although more research is needed to further explore the role of the MT_1_ receptor in melancholic depression, our findings suggest that the MT_1_ receptor may become a potential novel target for the therapeutics of endogenous depression, and selective MT_1_ agonists deserve to be tested as antidepressant drugs with chronobiotic effects.

## Supplementary Material

For supplementary material accompanying this paper, visit http://www.ijnp.oxfordjournals.org/

## Statement of Interest

The authors declare no conflicts of interest.
